# The Dispensary Chain in London

**Published:** 1916-12-09

**Authors:** Thomas Hayes

**Affiliations:** Clerk to the Governors. St. Bartholomew's Hospital, London, E.C.


*To the Editor of* The Hospital.

### "The Dispensary Chain in London."

*To the Editor of* The Hospital.

Sir,—In your issue of the 2nd instant, under the above heading, you allude to the agreement made between the governors of this hospital and the Corporation of the City of London for the establishment of a tuberculosis dispensary.

To avoid misapprehension, will you allow me to mention that, although the formal agreement between the two bodies has only just been sealed, the dispensary was actually opened on November 1, 1914, and up to the end of 1915 the attendances of patients had reached 2,322, the actual cost of working for the past two years being as follows: 1914-15, £603 10s. 2d.; 1915-16, £644 12s. 10d.?—I am, Sir, yours faithfully,

Thomas Hayes,
Clerk to the Governors.

St. Bartholomew's Hospital, London, E.C.,
December 4, 1916.

